# The *ABI4*-Induced Arabidopsis *ANAC060* Transcription Factor Attenuates ABA Signaling and Renders Seedlings Sugar Insensitive when Present in the Nucleus

**DOI:** 10.1371/journal.pgen.1004213

**Published:** 2014-03-13

**Authors:** Ping Li, Hua Zhou, Xiaoliang Shi, Bo Yu, Yan Zhou, Suli Chen, Yufeng Wang, Yu Peng, Rhonda C. Meyer, Sjef C. Smeekens, Sheng Teng

**Affiliations:** 1 Laboratory of Photosynthesis and Environmental Biology, Institute of Plant Physiology and Ecology, Shanghai Institutes for Biological Sciences, The Chinese Academy of Sciences, Shanghai, China; 2 Leibniz Institute of Plant Genetics and Crop Plant Research (IPK), Stadt Seeland OT Gatersleben, Germany; 3 Department of Molecular Plant Physiology, Utrecht University, Utrecht, The Netherlands; 4 Centre for BioSystems Genomics, Wageningen, The Netherlands; University of Toronto, Canada

## Abstract

Seedling establishment is inhibited on media containing high levels (∼6%) of glucose or fructose. Genetic loci that overcome the inhibition of seedling growth on high sugar have been identified using natural variation analysis and mutant selection, providing insight into sugar signaling pathways. In this study, a quantitative trait locus (QTL) analysis was performed for seedling sensitivity to high sugar in a Col/C24 F_2_ population of *Arabidopsis thaliana*. A glucose and fructose-sensing QTL, *GSQ11*, was mapped through selective genotyping and confirmed in near-isogenic lines in both Col and C24 backgrounds. Allelism tests and transgenic complementation showed that *GSQ11* lies within the *ANAC060* gene. The Col *ANAC060* allele confers sugar insensitivity and was dominant over the sugar-sensitive C24 allele. Genomic and mRNA analyses showed that a single-nucleotide polymorphism (SNP) in Col *ANAC060* affects the splicing patterns of *ANAC060* such that 20 additional nucleotides are present in the mRNA. The insertion created a stop codon, resulting in a truncated ANAC60 protein lacking the transmembrane domain (TMD) that is present in the C24 ANAC060 protein. The absence of the TMD results in the nuclear localization of ANAC060. The short version of the ANAC060 protein is found in ∼12% of natural Arabidopsis accessions. Glucose induces *GSQ11/ANAC060* expression in a process that requires abscisic acid (ABA) signaling. Chromatin immunoprecipitation-qPCR and transient expression analysis showed that ABI4 directly binds to the *GSQ11/ANAC060* promoter to activate transcription. Interestingly, Col *ANAC060* reduced ABA sensitivity and Glc-induced ABA accumulation, and *ABI4* expression was also reduced in Col *ANAC060* lines. Thus, the sugar-ABA signaling cascade induces *ANAC060* expression, but the truncated Col ANAC060 protein attenuates ABA induction and ABA signaling. This negative feedback from nuclear ANAC060 on ABA signaling results in sugar insensitivity.

## Author Summary

In plants, sugars function as signaling molecules that control important processes such as photosynthesis, growth, carbon distribution over different organs and the production of storage compounds. Sugar signaling requires the phytohormone abscisic acid (ABA) and the ABA-induced regulatory transcription factor *ABI4*. In this study, a genetic analysis identified the transcription factor *ANAC060* as an important component in establishing sugar sensitivity. It was found that, in natural *Arabidopsis thaliana* populations, the ANAC060 protein may occur as a long or a short version due to differential *ANAC060* mRNA splicing caused by a single-nucleotide polymorphism (SNP). The long ANAC060 protein with an intact transmembrane domain (TMD) is excluded from the nucleus, whereas the short version lacking the TMD is always present in the nucleus, where it regulates gene expression. Functional analyses indicated that Col *ANAC060* is involved in a novel negative feedback loop in the sugar-ABA signaling pathway. In this feedback loop model, *ABI4* activates *ANAC060* expression, but the nuclear presence of Col ANAC060 suppresses Glc-induced ABA accumulation and *ABI4* expression, thereby reducing responsiveness to sugar signals.

## Introduction

Plant growth and development depends on the energy and carbon building blocks provided by soluble sugars. For efficient carbon nutrient utilization, prokaryotic and eukaryotic organisms have evolved a range of sophisticated signal transduction pathways that link sugar status to growth and reproduction [1]–[3]. In plants, such sugar-sensing and -signaling systems regulate the expression of thousands of genes involved in the control of metabolic processes, growth, development and responses to the environment [4], [5]. Sugar signaling pathways closely interact with other signaling pathways, including those for light [6], phytohormones [7], stress [8], defense [9] and other nutrients, such as nitrogen and phosphate [10].

Seedling growth and greening is inhibited when high concentrations of sugars are added to the medium. In *Arabidopsis thaliana*, this phenomenon of early seedling development repression has been widely used as a convenient way of identifying mutants affected in sugar-sensing or signaling processes [11], [12]. Mutants that are insensitive or oversensitive to different sugars have been isolated in this way, and the genes involved have been identified. However, such genes function in a variety of biological processes [11]–[18], and it is thus challenging to understand how these genes participate specifically in sugar signaling pathways. Prominent among these genes are *HEXOKINASE1* (*HXK1*), which functions as a glucose (Glc) sensor [7], and genes involved in ABA and ethylene synthesis and signaling [11], [12], [17], [19]. The sugar-ABA cascade regulates many genes involved in photosynthesis and metabolism and is antagonized by ethylene signaling via the EIN3 protein [20], [21]. A central regulator in sugar-responsive gene expression in plants is the *ABI4* gene, which encodes an ERF/AP2 transcription factor [11], [13], [22]–[24]. ABI4 is a regulator of seed germination, plastid-to-nucleus signaling and photosynthesis, redox status, lipid biosynthesis and breakdown, lateral root development and cell wall modification (reviewed in [25], [26]). ABI4 is a versatile transcription factor, acting as both an activator and a repressor of gene expression. ABI4 binds directly to the identified ABI4 binding motif in the promoters of target genes [27]–[29].

Natural variation analysis provides an important tool for analyzing complex biological processes [30]. Previously, quantitative trait locus (QTL) mapping for seedling sugar sensitivity in L*er*/Cvi recombinant inbred line (RIL) populations led to the identification of several loci and genes controlling sugar sensitivity. For example, *Glc sensing QTL5* (*GSQ5*) [31] encodes the delay of germination 1(*DOG1*) gene, a major dormancy determinant. The sugar-ABA cascade induces *GSQ5/DOG1* expression, and the Cvi *GSQ5/DOG1* allele effectively enhances sugar-ABA signaling, thereby increasing sugar sensitivity [31]. Arabidopsis *fructose (Frc) sensing QTL6* (*FSQ6*) is another interesting QTL that is Frc specific, and the study of this QTL revealed an HXK1-independent Frc signaling pathway [32]. *FSQ6* encodes the *ANAC089* transcription factor, and the Cvi *ANAC089* allele specifies a dominant fructose insensitivity trait [32].

In this study, a novel sugar-sensing QTL (*GSQ11*) was identified in a Col X C24 F_2_ population using a selective genotyping approach. *GSQ11* associates with the segregation distortion region in highly Glc-insensitive F_2_ individuals, and the QTL was confirmed in near-isogenic lines (NILs). Further studies showed that *GSQ11* encodes the Arabidopsis NAC family transcription factor 060 (*ANAC060*). The Col *ANAC060* allele gene harbors a single-nucleotide polymorphism (SNP) that affects intron splicing, leading to a truncated protein that lacks the C-terminal membrane anchor domain. This truncated ANAC060 protein is constitutively located in the nucleus. *ANAC060* is likely a direct *ABI4* target, but, interestingly, the Col *ANAC060* allele attenuates sugar-induced ABA accumulation and renders seedlings insensitive to sugar.

## Results

### Selective genotyping identification of a Glc-sensing QTL in the Col X C24 F_2_ population

**Figure 1:**
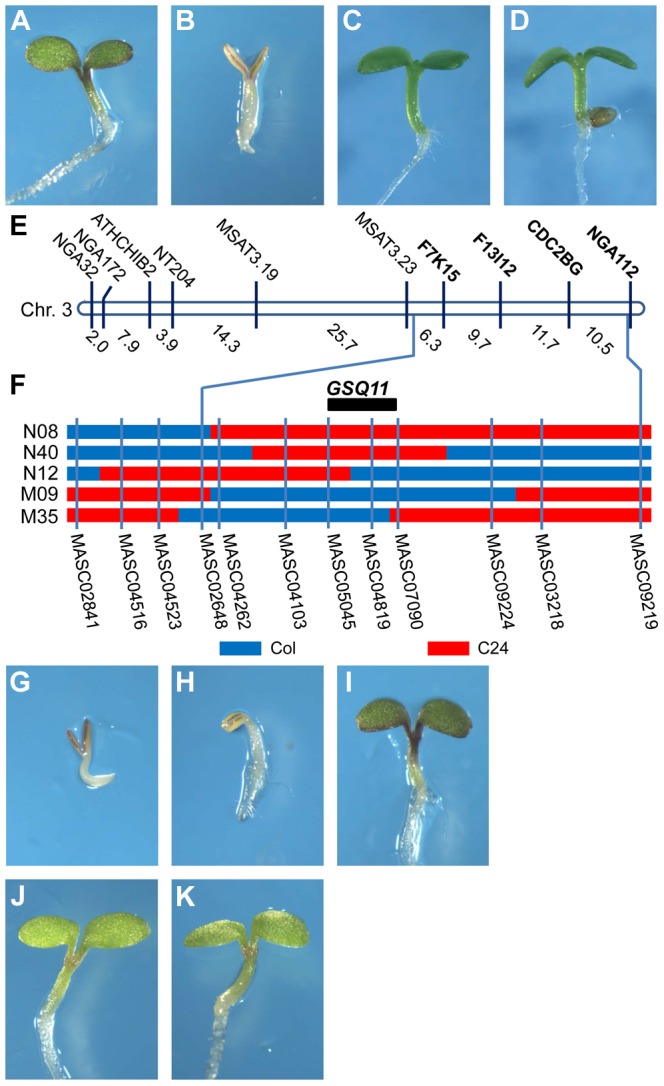
Identification of the Glc-sensing QTL *GSQ11* . Glc-sensitivity phenotypes of Col (A, C) and C24 (B, D) accessions. Seedlings were grown on agar-solidified 1/2 MS containing 5.5% Glc (A, B) or 5.5% Sor osmotic control (C, D) for 9 d at 22°C under continuous illumination. (E) Location of segregation distortion markers on chromosome 3. (F) Genotypes of the NILs and candidate region of *GSQ11*. Blue and red denote the Col and C24 accession genomic regions, respectively, (G–K) Glc sensitivities of the NILs (G) N08, (H) N40, (I) N12, (J) M09 and (K) M35 grown on agar-solidified 1/2 MS containing 5.5% glucose for 9 d at 22°C under continuous illumination.

The sugar sensitivity of Col and C24 seedlings was investigated on half-strength MS medium supplemented with increasing Glc concentrations, and it was found that C24 is substantially more sensitive to Glc than Col. On media supplemented with 5.5% Glc, the Col seedlings turned green and developed normally (Figure 1A), whereas the C24 seedlings were developmentally arrested and remained pale (Figure 1B). The growth of both accessions on 5.5% sorbitol (Sor) medium was not inhibited, showing that the sugar effect was not due to an osmotic effect (Figure 1C, D).

The selective genotyping strategy [33] was followed to identify QTLs for Glc sensitivity in the Col X C24 F_2_ population. Approximately 2000 F_2_ seeds were sown on 1/2 MS with 7% Glc, and 106 Glc insensitive seedlings were obtained. The genotypes of the insensitive F_2_ individuals were established using 81 polymorphic simple sequence length polymorphism (SSLP) markers (http://www.arabidopsis.org, http://www.inra.fr/vast/msat.php, http://amp.genomics.org.cn/). A segregation distortion region including SSLP markers F7K15, F13I12, CDC2BG and NGA112 was found on the long arm of chromosome 3 (Figure 1E, Table S1). The segregation ratios of these markers fit the 1∶2∶1 ratio in an unselected F_2_ population using 94 individuals (Table S1). Thus, it is likely that a Glc-sensing QTL (*GSQ*) is located in this region.

### Confirmation and fine mapping of the Glc-sensing QTL

Near-isogenic lines (NILs) were used to further study the *GSQ* identified on chromosome 3. NILs N08, N12 and N40 have single genomic regions from C24 integrated into the Col background ([34], Figure 1F). Conversely, NILs M35 and M09 have single genomic regions from Col integrated into the C24 background ([34], Figure 1F). These NILs were tested for seedling Glc sensitivity. Of the NILs with a Col background, N08 and N40 showed Glc-sensitive phenotypes (Figure 1G, H), whereas NIL N12 showed Glc insensitivity comparable to Col (Figure 1I). Of the NILs with a C24 background, both M35 and M09 showed Glc insensitivity compared to C24 (Figure 1J, K). These results confirm the presence of a Glc-sensing QTL in this genomic region, with the Col sequence presenting Glc insensitivity. The QTL was named *GSQ11*, and its confidence interval was deduced from the NIL genotypes and Glc-sensing phenotypes. *GSQ11* was localized to a 1.86-Mb region between markers MASC05045 (14.3 Mb) and MASC07090 (16.16 Mb) (Figure 1F).

### *GSQ11* encodes the transcription factor *ANAC060*

A total of 458 annotated genes are located in the *GSQ11* candidate region. Conspicuously present among these genes is *ANAC060* (*At3g44290*), which belongs to the *OsNAC8* subgroup of group I NAC transcription factors that includes *ANAC089* and *ANAC040* [35]. *ANAC089* was previously identified as determining seedling Frc sensitivity. The Cvi *ANAC089* allele specifically suppresses Frc signaling, allowing seedling development to proceed on high-fructose-containing media [32]. Therefore, the effect of *ANAC060* on Glc sensitivity and the possible association between *ANAC060* and *GSQ11* were investigated further.

**Figure 2:**
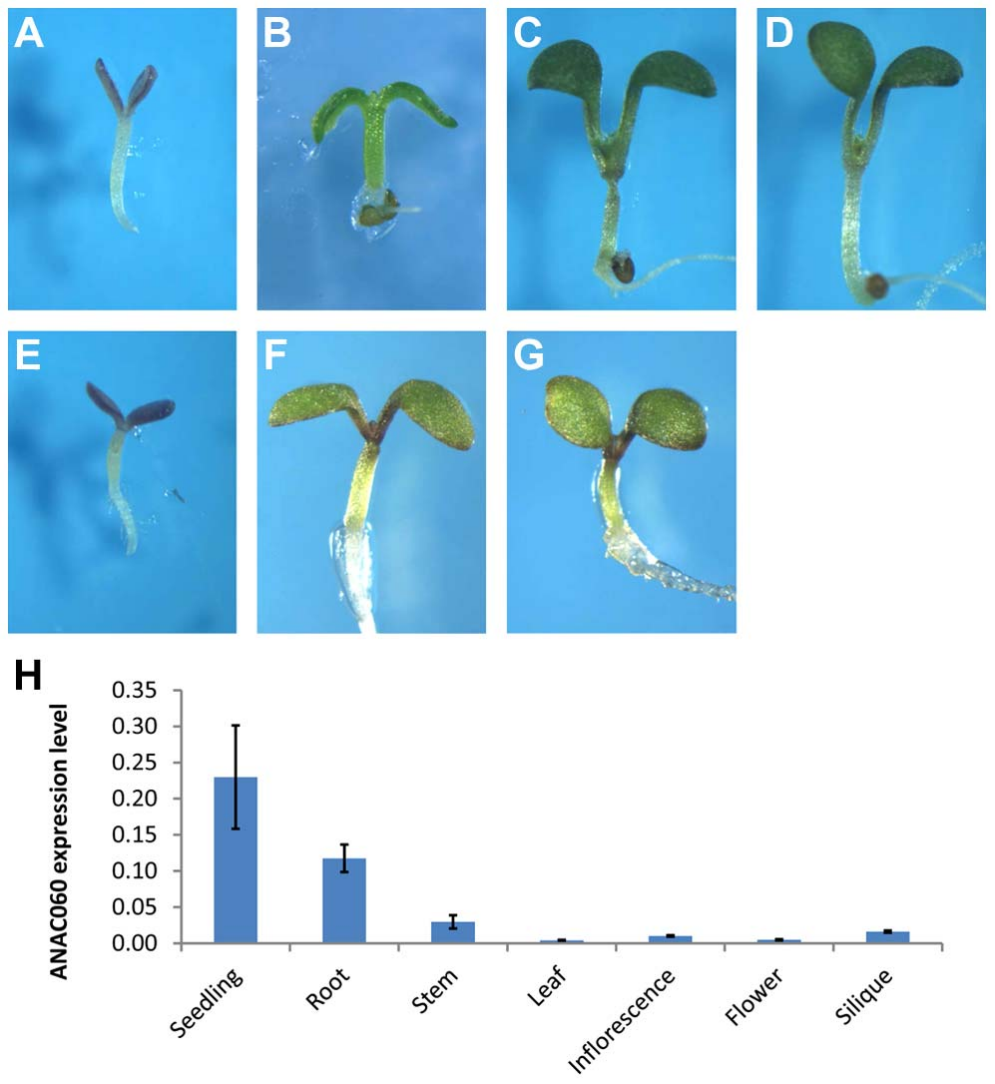
*GSQ11* encodes *ANAC060* . (A) Glc sensitivity of ***anac060*** **.** Mutant seedlings were grown on agar-solidified 1/2 MS containing 5.5% Glc for 9 d at 22°C under continuous light. (B) *anac060* growth on 5.5% Sor allows greening and development. Seedlings were grown on agar-solidified 1/2 MS containing 5.5% Sor for 9 d at 22°C under continuous light. (C–E) Allelism tests. Glc-sensitivity phenotypes of Col X *anac060* F_1_ (C), Col X N40 F_1_ (D) and NIL N40 X *anac060* F_1_ (E). Seedlings were grown on agar-solidified 1/2 MS containing 5.5% Glc for 9 d at 22°C under continuous light. (F–G) Transgenic complementation. (F) Glc sensitivity of the transgenic *anac060* mutant transformed with the Col *ANAC060* gene and (G) transgenic NIL N40 transformed with the Col *ANAC060* gene. Seedlings were grown on agar-solidified 1/2 MS containing 5.5% Glc for 9 d at 22°C under continuous light. (H) *ANAC060* expression levels in 7-day-old seedlings and in different organs of 30-day-old plants.

A T-DNA insertion mutant of *anac060* (Salk_012554C, Col background), in which a T-DNA insert is present in the second intron of the gene, was obtained (Figure S1A), and the insertion was confirmed by a PCR analysis (Figure S1B). No *ANAC060* mRNA could be detected by RT-PCR in this mutant (Figure S1C). Interestingly, the growth of *anac060* on 5.5% Glc resulted in seedling developmental arrest (Figure 2A), whereas Col is Glc insensitive. The Glc-sensitive phenotype of *anac060* was found not to be the result of osmotic stress, as indicated by normal growth on 5.5% Sor (Figure 2B).

Allelism tests for *GSQ11* and *ANAC060* were performed, and the F_1_ seedlings of Col X *anac060* and Col X N40 were both found to be insensitive to 5.5% Glc, similar to Col. This finding further indicated that both the C24 *GSQ11* allele and *anac60* are recessive (Figure 2C, D). Importantly, the F_1_ seedlings of the *anac060* X N40 cross were found to be Glc sensitive, as are the N40 and *anac060* parents (Figure 2E), strongly suggesting that *GSQ11* and *ANAC060* are allelic.

*GSQ11* and *ANAC060* allelism was confirmed by transferring the Col *ANAC060* gene into N40 and *anac060* using the floral dip transformation method. Seedlings of the N40 and *anac060* lines transformed with the Col *ANAC060* gene were Glc insensitive, unlike the Glc-sensitive N40 and *anac060* parents (Figure 2F, G). These results identify *ANAC060* as *GSQ11*.

The Frc sensitivities of *anac060* and of *anac060* transformed with Col *ANAC060* were then investigated. Seedling development of *anac060* was arrested on media containing 6.5% Frc (Figure S2B), whereas the seedlings of *anac60* transformed with Col *ANAC060* and of wild-type Col were green and developed normally (Figure S2A, C). The Frc-sensitive phenotype of the *anac060* seedlings also reverted to Frc insensitivity in *anac060* harboring the Col *ANAC060* transgene (Figure S2C). These results show that *ANAC060* is involved in both Glc and Frc signaling.

*ANAC060* expression levels were determined in different plant tissues by qPCR. *ANAC060* was found to be highly expressed in both seedlings and roots and weakly expressed in the leaf, stem, flower and silique (Figure 2H).

### Differential mRNA splicing renders Col *GSQ11/ANAC060* insensitive to Glc

**Figure 3:**
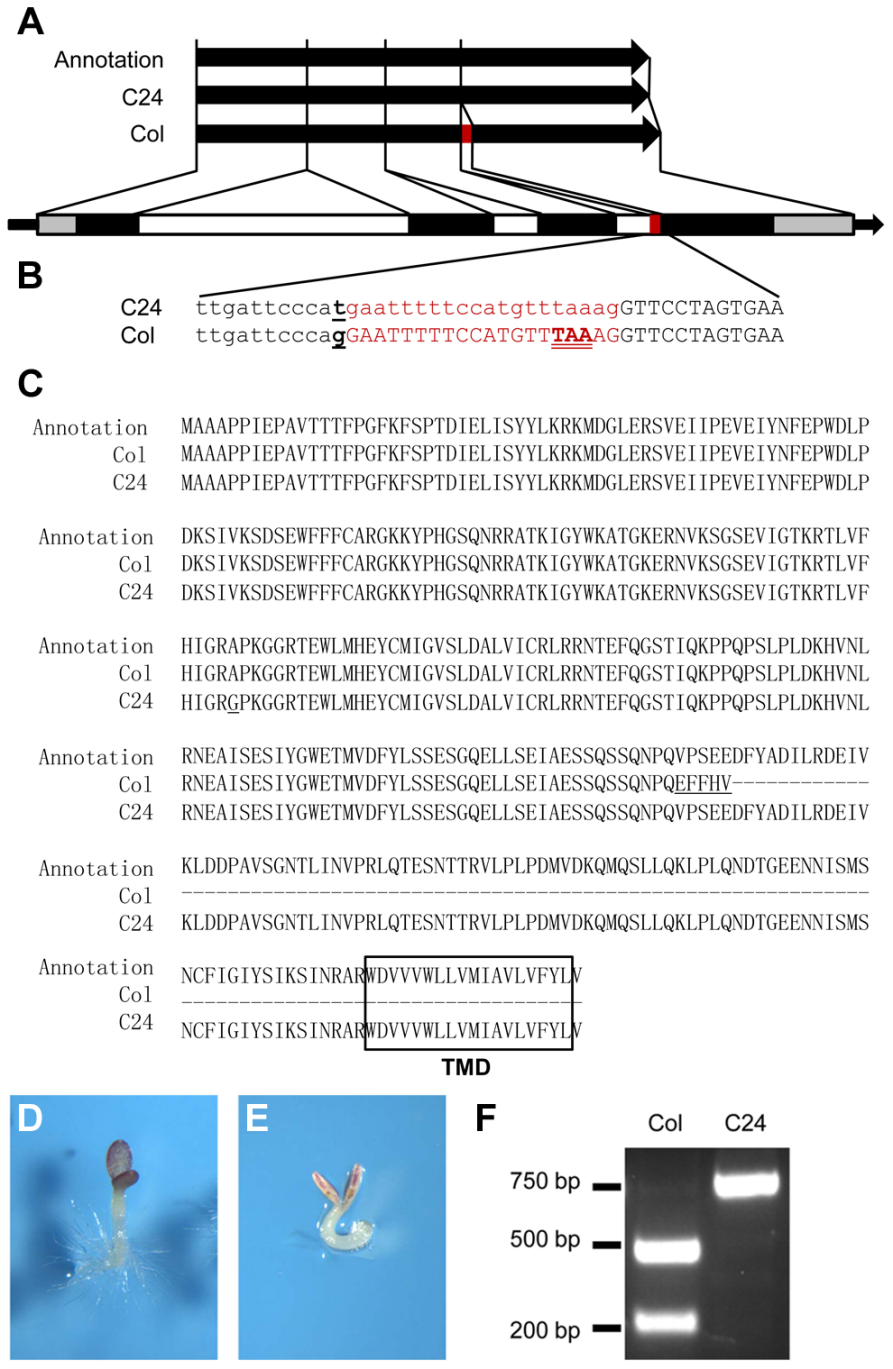
A QTN alters the splicing pattern of Col and C24 *ANAC060* mRNAs. (A and B) Splicing patterns of Col and C24 *ANAC060* mRNA compared to the TAIR annotation. The red bar indicates the extra 20 nucleotides in the Col accession. The black and open bars indicate the exons and introns, respectively. The grey bars represent the 5′ and 3′ UTRs. (B) Details of the SNP and splicing patterns. The underlined letter indicates the SNP. The red print indicates the sequence of the 20-nucleotide fragment that is absent (small caps) in the C24 mRNA and present (large caps) in the Col mRNA. The in-frame TAA stop codon in Col is double underlined. (C) Amino acid sequences of the TAIR annotation (top), Col (middle) and C24 (bottom) GSQ11/ANAC060 proteins. The transmembrane domain (TMD) is boxed. The underlined letters indicate the amino acid changes compared to the TAIR annotation. (D–E) Glc sensitivities of transgenic *anac060* (D) and N40 (E) transformed with the mutated Col *ANAC060* ^G-T^ gene. Seedlings were grown on agar-solidified 1/2 MS containing 5.5% Glc for 9 days at 22°C under continuous illumination. (F) The CAPS marker for detecting the QTN between Col and C24. The primer sequences were CAPS-F 5′ GGAAAGCCACAGGAAAAGAGC 3′ and CAPS-R 5′ CACCAACACGGCTATCATAACGAG 3′. The Col-type alleles are digested by ScrFI, whereas the Cvi-type alleles are not.

*ANAC060* is annotated as a NAC transcription factor with a transmembrane domain (TMD) and was named NAC with transmembrane motif 1-like 5 (*NTL5*) [36]. The TAIR (www.arabidopsis.org)-annotated ANAC060 protein consists of 335 amino acids with a C-terminally located TMD. Remarkably, the Col *ANAC060* full-length cDNA clone GSLTLS7ZC02 (www.ncbi.nlm.nih.gov/nucleotide/BX823820) encodes a protein of 228 amino acids that lacks the TMD. In yeast, the Col ANAC060 protein fused to the GAL4 DNA-binding protein strongly activated a GAL4 UAS promoter-containing reporter gene (Figure S3), suggesting that ANAC060 functions as a transcriptional activator. Serial deletions of the Col ANAC060 protein were tested for transcriptional activity in yeast, resulting in the identification of a region including V^196^ D^197^ F^198^ that is conserved in ANAC060 and ANAC089 as essential for transcriptional activity (Figure S3). An alignment analysis showed that, in the Col *ANAC060* mRNA coding sequence, 20 base pairs (bp) are inserted that include an in-frame TAA stop codon, explaining the Col ANAC060 protein truncation. These 20 bp are absent in the C24 *ANAC060* mRNA. It appears that the Col and C24 *ANAC060* mRNAs are spliced differently (Figure 3A).

Comparing the genomic sequences of the Col and C24 *ANAC060* genes revealed the probable cause for the differential splicing. An SNP is located precisely at the 3′ end of the 3rd intron splicing acceptor site of the Col *ANAC060* gene. In Col, a G nucleotide is located at this position, whereas this is a T in C24 (Figure 3B). This G to T transversion affects the splicing pattern: a functional AG acceptor splice site is present in Col and a non-functional AT site is present in C24 (Figure 3B). However, the annotation in the TAIR database does not include this AG acceptor splice site, producing the 335-amino acid-long version of the ANAC60 protein that includes the TMD (Figure 3C).

The association between the *ANAC060* splicing pattern and Glc sensitivity was illustrated by introducing a G to T point mutation in the Col *ANAC060* gene and transforming this mutated Col gene into *anac060* and NIL N40. cDNA derived from the mRNA of transgenic *anac060* harboring the Col *ANAC060^G-T^* construct was sequenced. Importantly, the Col *ANAC060^G-T^* lines showed the same splicing pattern as C24 (Figure S4A). The splicing pattern in the transgenic *anac060* lines transformed with the wild-type Col *ANAC060* gene was identical to that of the Col accession (Figure S4B). As expected, the introduced mutated *ANAC060^G-T^* gene did not complement the Glc-sensitivity phenotypes of *anac060* and NIL N40 (Figure 3D, E). These results confirm that the G-T SNP is responsible for the differential splicing in Col and C24 and establishes this SNP as a quantitative trait nucleotide (QTN) for sugar sensitivity.

The frequency of the natural G-T substitution causing the Glc-sensitive phenotype was investigated in Arabidopsis accessions. A cleaved amplified polymorphic sequence (CAPS) marker that specifically detects this SNP was developed (Figure 3F), and the haplotypes of 66 accessions were established. Six of the 66 accessions tested were of the Col type, and the remaining 60 accessions were of the C24 type (Table S2). The *ANAC060* SNP was further analyzed using public data on 507 resequenced Arabidopsis accessions (http://signal.salk.edu/atg1001/3.0/gebrowser.php). The haplotypes of 436 accessions were T, including C24; those of 69 accessions were G, including Col (Table S3). Accessions Got-22 and Got-7 have a C nucleotide at the splice site position, differing from both Col and C24.

### Differential localization of the Col and C24 ANAC060 proteins

**Figure 4:**
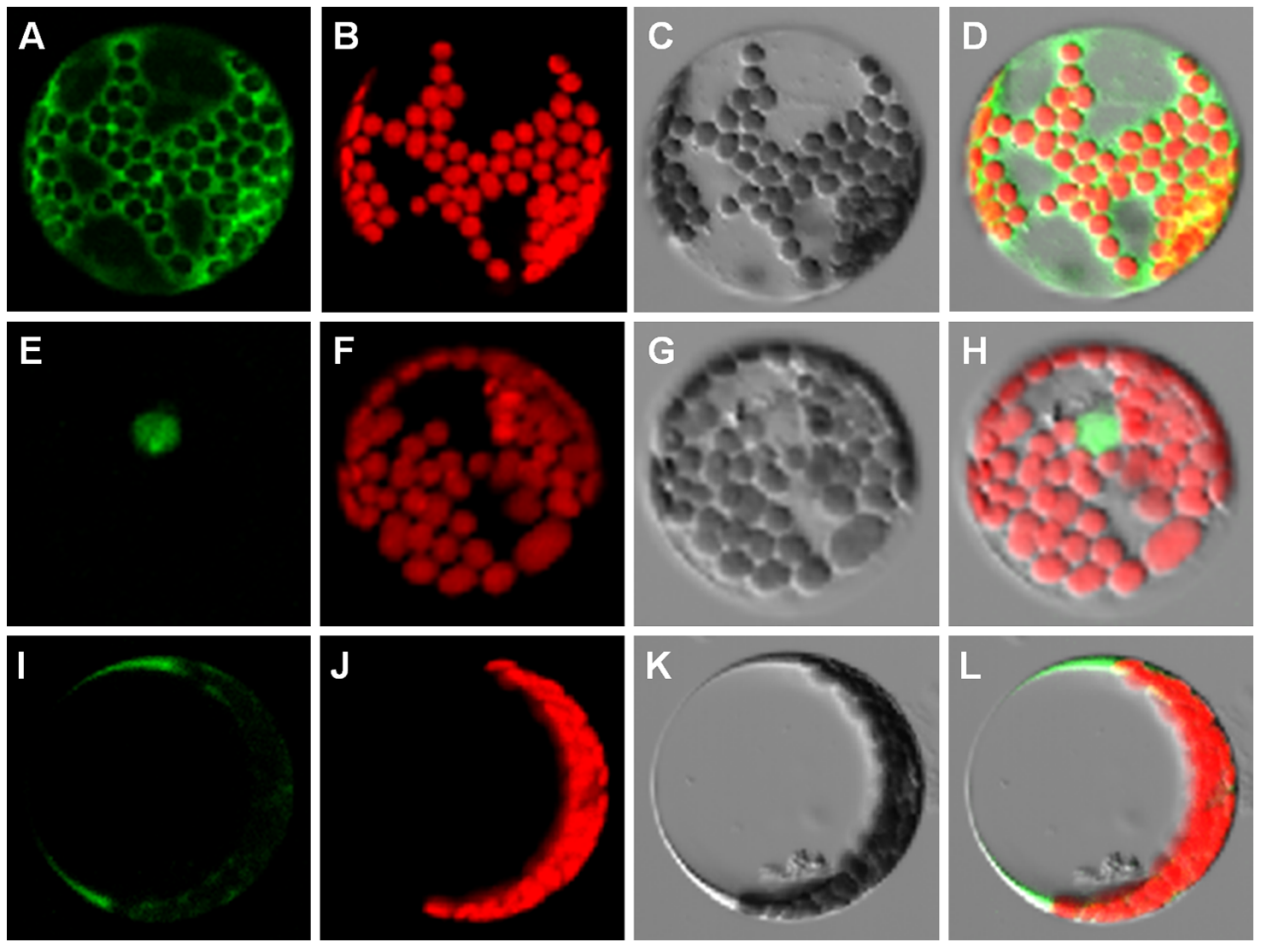
Localization of the Col and C24 ANAC060 proteins. (A–D) Localization of GFP expressed from the control vector PA7, (A) GFP fluorescence image, (B) chloroplast fluorescence, (C) bright-field image and (D) merged image of A–C. (E–H) Col GFP::ANAC060 fusion protein localization, (E) GFP fluorescence, (F) chloroplast fluorescence, (G) bright-field image and (H) merged image of E–G. (I–L) C24 GFP::ANAC060 fusion protein localization, (I) GFP fluorescence, (J) chloroplast fluorescence, (K) bright-field image and (L) merged image of I–K.

The absence of a TMD in the truncated Col-type ANAC060 protein most likely results in differential protein localization compared to the C24-type ANAC060 protein, which includes the TMD. Differential localization of Col and C24 ANAC060 proteins was investigated by fusing these proteins N-terminally to GFP, followed by transient expression in Arabidopsis mesophyll protoplasts using the PA7 vector (created by Dr. Katrin Czempinski, Potsdam University, Germany). As expected, the PA7 vector expressing only the GFP protein was localized by confocal laser-scanning microscopy in the cytosol and nucleus (Figure 4A–D). Interestingly, the Col GFP::ANAC060 fusion protein was located exclusively in the nucleus (Figure 4E–H), whereas the C24 GFP::ANAC060 fusion protein was located in the cytosol (Figure 4I–L). Therefore, the presence of the TMD effectively retains the C24 ANAC060 protein in the cytosol, most likely in association with endomembranes [37].

### Glc induction of *ANAC060* requires ABA signaling

**Figure 5:**
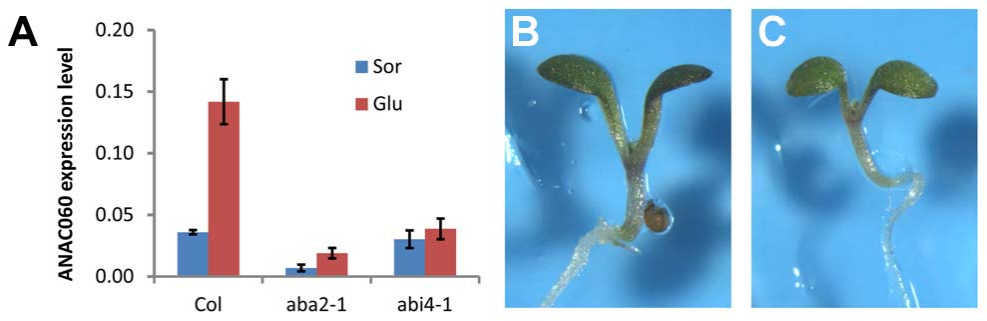
Glc induction of *ANAC060* requires ABA signaling. (A) *ANAC060* mRNA levels in Col wild-type, *aba2-1* and *abi4-1* mutants grown at 22°C under continuous illumination for 7 days in 5.5% Glc or 5.5% Sor. The values represent the average of three technical repeats from a representative experiment. The bars indicate the standard errors. Similar results were obtained in three independent experiments. (B) Glc sensitivities of the *anac060 aba2* double mutant and (C) *anac060 abi4* double mutant. Seedlings were grown on agar-solidified 1/2 MS containing 5.5% Glc for 9 d at 22°C under continuous light.

The Glc responsiveness of *ANAC060* was investigated, and it was found that this gene is strongly induced by Glc (Figure 5A). Many Glc-responsive genes depend on ABA synthesis and signaling. Interestingly, *ANAC060* does not respond to Glc in the *aba2-1* ABA-deficient mutant and the *abi4-1* ABA signaling mutant (Figure 5A). Thus, *ANAC060* is responsive to the sugar-ABA signaling cascade. The genetic interaction between the ABA signaling pathway and *GSQ11/ANAC060* in determining Glc sensitivity was investigated in *anac060 aba2* and *anac060 abi4* double mutants. Both the *anac060 aba2* and *anac060 abi4* double mutants showed sugar-insensitive phenotypes similar to the *aba2* and *abi4* single mutants (Figure 5B, C). These results show that *ABA2* and *ABI4* are epistatic to *GSQ11/ANAC060*.

### ABI4 binds to and activates the *ANAC060* promoter

**Figure 6:**
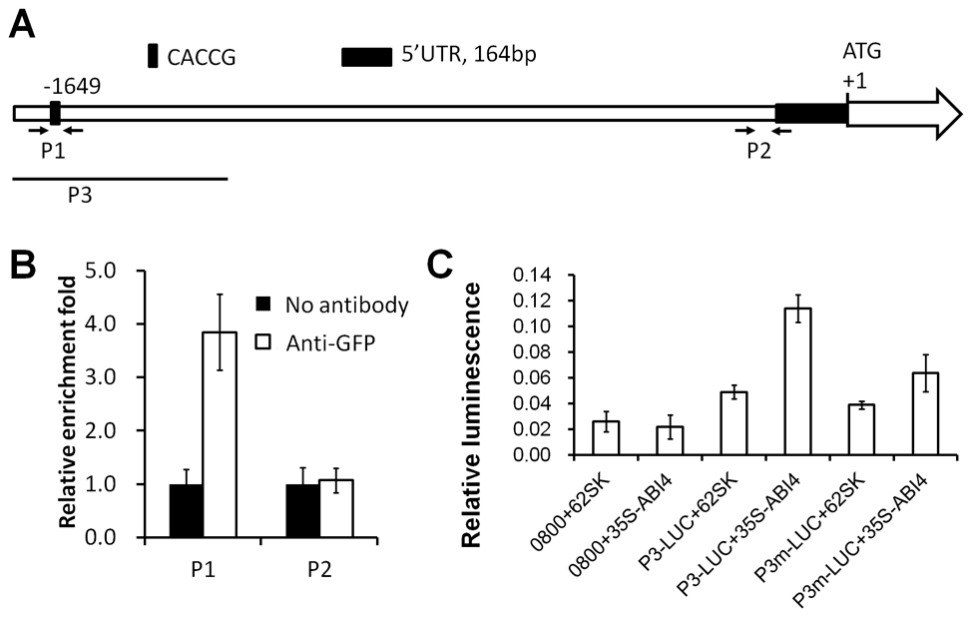
ABI4 directly activates *ANAC060*. (A) The *ANAC060* promoter region: P1 indicates the CACCG motif bound by ABI4, and P2 is proximal to the transcription initiation site. The arrows indicate the primers used for the P1 and P2 region ChIP-qPCR analyses. P3 indicates the fragment used in the transient expression assays. (B) P1 and P2 promoter fragment enrichment following ChIP-qPCR performed in the absence (No antibody) or presence (Anti-GFP) of anti-GFP antibodies. (C) Protoplast transient expression assay using the *ANAC060* P3 promoter fragment (A). The luciferase luminescence intensity was quantitated following transfection with different vectors. 0800+62SK indicates protoplast transfection with the empty pGreenII 0800-LUC and pGreenII 62-SK vectors. 0800+35S-ABI4 indicates protoplast transfection with the empty pGreenII 0800-LUC vector and pGreenII *35S-ABI4*. P3-LUC+62SK indicates protoplast transfection with pGreenII with the *ANAC060* P3 promoter fragment fused to LUC and the empty pGreenII 62-SK vector. P3-LUC+35S-ABI4 indicates protoplast transfection with pGreenII with the *ANAC060* P3 promoter fragment fused to LUC and pGreenII *35S-ABI4*. P3m-LUC+62SK indicates protoplast transfection of pGreenII with the mutated (CACCG to TTTAA) *ANAC060* P3 promoter fragment fused to LUC and the empty pGreenII 62-SK vector. P3m-LUC+35S-ABI4 indicates protoplast transfection with pGreenII with the mutated (CACCG to TTTAA) *ANAC060* P3 promoter fragment fused to LUC and pGreenII 35S-ABI4. Three biological repeats were performed, and cotransfection with *35S-Renilla LUC* was used for normalization.

ABI4 regulates many sugar-responsive genes by direct promoter binding via the ABI4 binding motif [28]. In *ANAC060*, a CACCG ABI4 binding motif is located 1649 bp upstream of the ATG initiation codon (Figure 6A), suggesting that ABI4 induces *ANAC060* by directly binding to the promoter. Accordingly, direct ABI4 binding to the *ANAC060* promoter was investigated using ChIP (chromatin immunoprecipitation)-qPCR in a *35S-ABI4::GFP* transgenic line (Col accession) [38]. This transgenic line showed a Glc-sensitive phenotype, indicating that the ABI4::GFP fusion protein retained its biological function (Figure S5A–B). The *ANAC060* mRNA level in this ABI4-overexpressing line grown on glucose was approximately 5-fold higher than in wild-type (). Fragmented chromatin was immunoprecipitated using an anti-GFP antibody, and *ANAC060* promoter fragments were quantitated using qPCR. In these experiments, a close to four-fold enrichment was observed for the CACCG-containing region (P1) in the *ANAC060* promoter relative to the control; no such enrichment was found in a control region (P2) of the promoter (Figure 6B). Thus, it is likely that ABI4 binds directly to the CACCG region of the *ANAC060* promoter in vivo.

The regulation of *ANAC060* expression by ABI4 was investigated using Arabidopsis mesophyll protoplasts and the dual luciferase transient expression system. A luciferase reporter plasmid was constructed containing the P3 part of the *ANAC060* promoter fused to LUC (*P3-LUC*) in the pGreenII vector (Figure 6A). High relative luciferase activity was detected only when the reporter construct was co-transfected with the *35S-ABI4* construct in the pGreenII vector (*35S-ABI4*) (Figure 6C). The transfection of vectors lacking either *35S-ABI4* or the P3 target promoter element resulted in low relative luciferase activity comparable to single transfections of *35S-ABI4* or *P3-LUC* (Figure 6C). The role of the CACCG ABI4 binding motif in *ANAC060* promoter activation was investigated by mutational analysis. Cotransfection of *35S-ABI4* and the P3 element harboring a mutated CACCG sequence (*P3m-LUC*) caused reduced luciferase activity compared to the wild-type *P3-LUC* sequence (Figure 6C). These findings, together with the ChIP-qPCR results, support the conclusion that ABI4 induces *ANAC060* transcription in vivo.

### *ANAC060* attenuates Glc-induced ABA accumulation and ABA sensitivity

**Figure 7:**
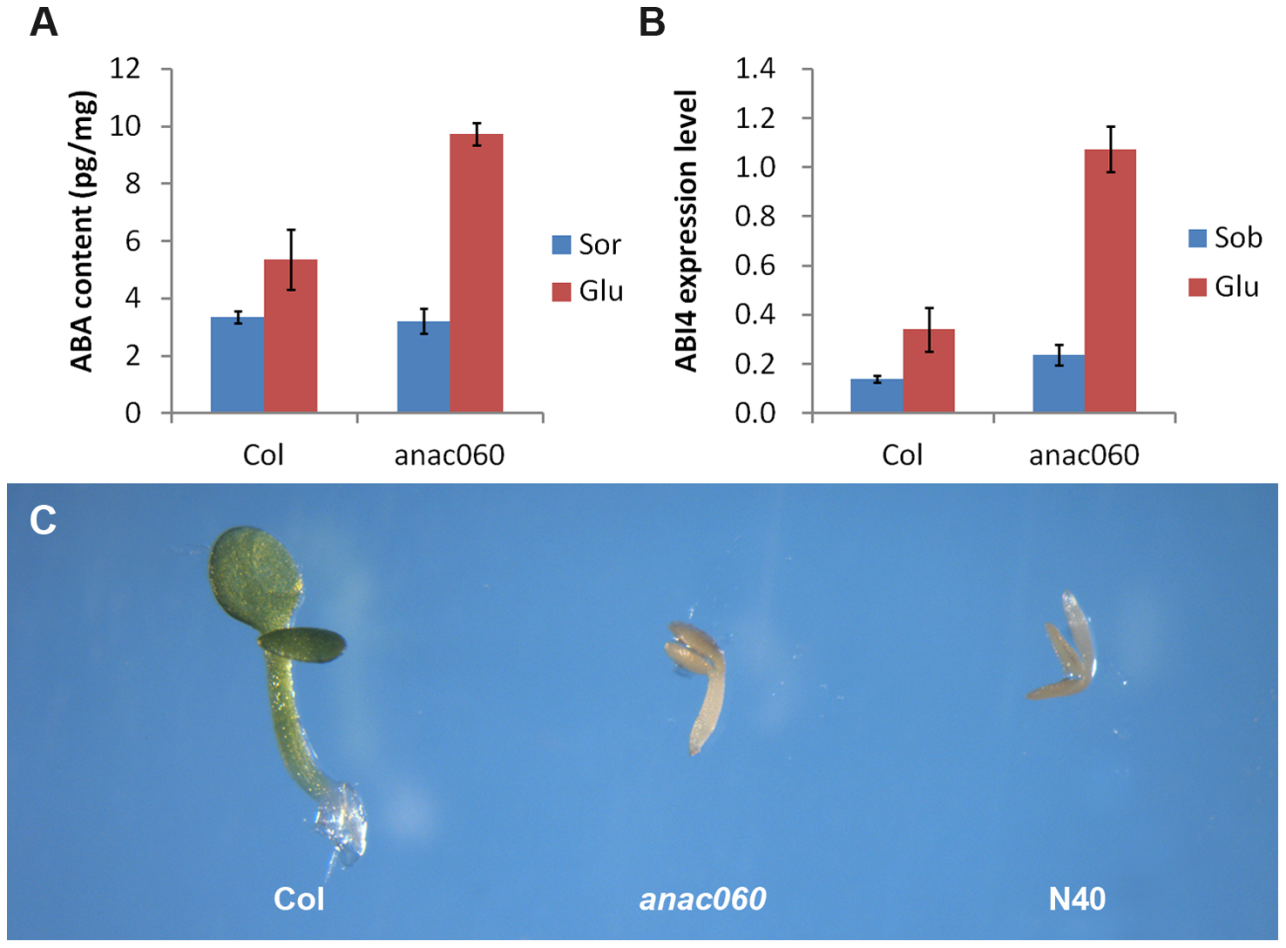
*ANAC060* attenuates Glc-induced ABA accumulation and ABA sensitivity. (A) ABA content of Col and *anac060* seedlings grown at 22°C under continuous illumination for 5 days on 5.5% Glc or 5.5% Sor. (B) *ABI4* expression level of Col and *anac060* seedlings grown at 22°C under continuous illumination for 5 days on 5.5% Glc or 5.5% Sor. (C) Col, *anac060* and NIL N40 seedlings grown on 1/2 MS with 0.5 µmol/L ABA for 5 days under continuous illumination.

ABA levels were quantitated in 5-day-old Col and *anac060* seedlings grown on 5.5% Glc or on a Sor osmotic control. Growth on Glc increased the ABA content both in Col and *anac060* (Figure 7A) compared to the osmotic control. Interestingly, the ABA levels in the Glc-grown *anac060* seedlings were ∼twofold higher than in Col (Figure 7A). As expected, Glc induced *ABI4* expression in Col, but a nearly fourfold higher *ABI4* mRNA level was detected in *anac060* (Figure 7B). The increases in ABA and *ABI4* expression in *anac060* likely explain the *anac060* Glc-sensitive phenotype.

ABA sensitivity was investigated in Col, *anac060* and NIL N40 in the absence of added sugars. Supplementing the 1/2 MS growth medium with 0.5 µmol/L ABA repressed seedling development in *anac060* and NIL N40 compared to a control medium, whereas Col seedling development was unaffected (Figure 7C, Figure S6). These results indicate that the Col *ANAC060* reduces sensitivity to ABA.

## Discussion

The preparation of RIL populations for QTL mapping is laborious and time consuming. In contrast, selective genotyping represents a rapid and convenient way to locate QTLs [33], [39]. Selective genotyping has been used to map QTLs in maize [40], wheat [41], barley [42], tomato [43]–[45] and rat [46]. Glc sensitivities of C24 and Col accessions were compared, and it was found that C24 is substantially more sensitive to Glc than is Col. Glc insensitivity was investigated in Col X C24 F_2_ individuals, and a segregation distortion region was identified. The segregation distortion-associated QTL, *GSQ11*, was confirmed in NIL lines with Col and C24 backgrounds.

Previously, QTL analysis for sugar sensitivity was undertaken in the L*er*/Cvi RIL population, and nine QTLs specifying Glc or Frc sensitivity were identified [31], [32]. The *GSQ11* locus identified here represents a novel locus not previously observed in the L*er*/Cvi RIL population. *GSQ11* is identical to *ANAC060*, a novel gene that determines sugar sensitivity. In natural populations, *ANAC060* is present in two variants: the Col variant, encoding a 228-amino acid-long protein that lacks the TMD, and a C24 variant encoding a 335-amino acid-long protein that includes the TMD. In the Col *ANAC060* allele, an SNP creates a functional AG splice acceptor site, resulting in differential splicing that introduces a premature stop codon; conversely, an AT sequence is present at this location in C24, resulting in normal splicing and a full-length protein. As a consequence, the Col protein is constitutively localized to the nucleus, whereas the C24 protein is retained in the cytosol by its TMD.

Membrane-tethered transcription factors (MTTFs) are found in virtually all major transcription factor families in plants [47]. The TMDs of MTTFs are essential for membrane insertion, thereby excluding the proteins from the nucleus. However, developmental, metabolic and environmental stimuli can induce proteolytic cleavage of the TMD in MTTFs, resulting in their nuclear migration, where they can regulate target genes [48]. This mechanism allows for rapid responses to changing conditions. For example, membrane-attached ANAC089 TMD cleavage is controlled by the cellular redox status, and reducing conditions promote the nuclear migration of this protein [37]. At least 18 NAC transcription factors with TMDs are encoded by the Arabidopsis genome, including *ANAC060* and *ANAC089* [47]. Previously, a natural variation analysis identified the Cvi allele of the *ANAC089/FSQ6* transcription factor as promoting seedling Frc insensitivity. This Cvi *ANAC089* allele lacks a TMD, similar to Col *ANAC060*, and the truncated Cvi ANAC089 protein is also localized to the nucleus. However, truncated ANAC089 is specifically Frc insensitive, whereas truncated ANAC060 suppresses the response to both Glc and Frc. Further investigations are needed to understand the mechanism by which nuclear-localized ANAC060 and ANAC089 proteins establish different responses to sugars and the role of ABA signaling in this process. Such investigations include the identification of genes differentially regulated by Glc and Frc in variants of ANAC060 and ANAC089.

Glc induces *GSQ11/ANAC060* expression, and this induction requires an intact ABA signaling pathway. The Glc sensitivity of the *anac060* mutant depends on an intact ABA signaling pathway, and *anac060* becomes Glc insensitive when combined with ABA biosynthesis (*aba2*) or signaling (*abi4*) mutants. The ABA-induced transcription factor *ABI4* is a central regulator of sugar-responsive gene expression in plants. ABI4 induces *ANAC060* expression by interacting with the ABI4 binding motif in the *ANAC060* promoter. Two- to threefold higher Glc-induced ABA accumulation was reported in the Glc-treated *gin6/abi4* mutant [13], suggesting that ABI4 induction inhibits ABA accumulation in the sugar-ABA signaling pathway. In the present study, enhanced sugar-induced ABA accumulation was also observed in the *anac060* mutant. Therefore, ANAC060 localized to the nucleus also appears to be part of a negative feedback loop in the ABA-mediated sugar signaling pathway. The sugar-ABA signaling pathway induces the expression of *ANAC060* through *ABI4*, whereas the nuclear presence of the ANAC060 protein represses sugar-ABA signaling, rendering seedlings insensitive to sugars.

## Materials and Methods

### Plant materials

The F_2_ population derived from a cross between Col and C24 was used for the QTL analysis. N08, N12 and N40 are NILs with single genomic regions of C24 integrated into the Col background; M35 and M09 are NILs with single genomic regions of Col integrated into the C24 background [34]. The *anac060* T-DNA insertion mutant, Salk_012554C, was obtained from ABRC. *35S-ABI4::GFP* transgenic line was provided by Dr. Xie [38].

### Analysis of sugar sensitivity and ABA sensitivity

Sterilized seeds were placed on 0.1% agarose for 4 days at 4°C in the dark for stratification and then plated on solidified 1/2 MS medium, pH 5.8. Sugars, sorbitol and ABA were added at the concentrations indicated. The plates were incubated at 22°C under continuous fluorescent light for 9 days, and the sugar- and ABA-sensitivity phenotypes were scored.

### QTL analysis by selective genotyping

A total of 106 Col X C24 F_2_ individuals insensitive to 7% Glc were selected and genotyped together with an unselected F_2_ population of 94 individuals. Eighty-one polymorphic SSLP markers between Col and C24 were used for genotyping (http://www.arabidopsis.org, http://www.inra.fr/vast/msat.php, http://amp.genomics.org.cn/). The statistical approach for QTL identification was as previously described [44].

### Constructs

The promoter (1794 bp upstream of the start codon) and coding regions (2111 bp) of *ANAC060* were amplified separately from Col genomic DNA using the primers presented in Table S4 and individually cloned into the pCAMBIA1301 to construct complementation vectors. The G-T point mutation was introduced into the complementation vector using the QuikChange Multi Site-Directed Mutagenesis Kit (Stratagene). The coding sequences of the *ANAC060* gene from C24 (1028 bp) and Col (687 bp) were amplified from cDNA using the primers presented in Table S5 and subcloned into PA7 to construct vectors for the protein localization assay in protoplasts.

### Transactivation activity assay in yeast

The transactivation activity assays in yeast were performed using the Matchmaker GAL4 Two-Hybrid System 3 (Clontech), with modifications as described [49]. The full-length *ANAC060* cDNA (clone GSLTLS7ZC02) was used to create *pANAC060ΔC1*, *pANAC060ΔC2* and *pANAC060ΔC3* by PCR amplification using the primers presented in Table S6. The PCR products were digested with BamHI and PstI and inserted into PGBKT7, resulting in an in-frame fusion of *ANAC060* sequences with the *GAL4* DNA-binding domain. The plasmids were transformed into yeast strain PJ69-4A, and the yeast cultures were grown overnight in liquid medium and diluted to an OD600 of 0.5. Serially dilutions were dropped onto either -Trp SD media or -Trp/-His/-Ade SD media.

### ChIP-qPCR

Seeds of Col and the *35S-ABI4::GFP* transgenic line were grown on 1/2 MS medium for approximately 2 weeks. Seedlings (3 g) were harvested and crosslinked with 1% formaldehyde for 15 minutes under vacuum; the crosslinking was stopped by the addition of 0.125 M glycine. The seedlings were homogenized in liquid nitrogen, and nuclei were isolated. The isolated nuclei were treated with ultrasonic waves (Bioruptor UCD-200) to fragment the chromatin into 200–300-bp fragments. Immunoprecipitations were performed with an anti-GFP antibody (Abcam) and protein A beads (Millipore). Mock immunoprecipitations in the absence of anti-GFP served as controls. DNA was precipitated by isopropanol, washed with 70% ethanol and dissolved in 35 µl water containing 10 µg/µl RNase. The qPCR analysis was performed using primers (Table S7) corresponding to different promoter regions of *ANAC060*. The relative enrichment of each fragment was calculated by normalizing the values for transgenic plants against the values for wild-type, as described [50].

### Dual luciferase essay

The Arabidopsis protoplast isolation and transfection followed the protocols of Dr. Sheen's laboratory [51]. The P3 region of the *ANAC060* promoter was amplified (Table S8) and cloned into the pGreenII *0800-LUC* (firefly luciferase) vector [52] to construct the pGreenII *P3-LUC* vector. The mutated P3 fragment (P3m) was artificially synthesized (Shanghai Generay Biotech Co., Ltd.) and cloned into the pGreenII *0800-LUC* vector, similar to the P3 fragment. The *ABI4* cDNA was fused to the pGreenII 62-SK vector [52] to construct the pGreenII *35S-ABI4* vector. In each co-transfection assay, 6 µg pGreenII *P3-LUC* plasmid and 12 µg *35S-ABI4* plasmid were used. Protoplasts that had been incubated for 16 h were subjected to luciferase assays using the Promega dual-luciferase reporter assay system and the GloMax 20/20 luminometer.

### Real-time RT-PCR

Total RNA was isolated using the Plant RNA Kit from Yuanpinghao Bio (www.yph-bio.com). Aliquots (1 µg) were reverse transcribed using TransScript One-Step gDNA Removal and cDNA Synthesis SuperMix (TransGen Biotech). Five µl of cDNA was used per real-time PCR reaction with 20 µl GoTaq q-PCR Master Mix from Promega (http://www.promega.com) and 0.8 µl of primers diluted to 10 pM (Table S9). PCR was performed using the Roche LightCycler 96 SW 1.0 Real-Time PCR system sequence detector. The expression levels of the *ANAC060* and *ABI4* genes were calculated relative to the *PP2A* [53] levels using the Q-gene method, which takes the relative efficiencies of the different primer pairs into account [54], [55]. The sequences of the primers used for the amplification of *ANAC060* and *ABI4* are presented in Table S9.

### Quantification of endogenous ABA

Seedlings were ground in liquid nitrogen, and 150 mg of the powder was homogenized and extracted for 24 h in methanol containing D6-ABA (OIChemIm Co. Ltd.) as an internal standard. ABA was purified using an Oasis Max solid-phase extraction cartridge (150 mg/6 cc; Waters) and eluted with 5% formic acid in methanol. The eluate was dried, and ABA was quantitated as described [38] using a liquid chromatography–tandem mass spectrometry system with an Acquity ultra-performance liquid chromatograph (Acquity UPLC; Waters) and a triple quadruple tandem mass spectrometer (Quattro Premier XE; Waters). Three biological replications were performed for each treatment.

## Supporting Information

Figure S1 Confirmation of the *anac060* T-DNA insertion mutant (Salk_012554C). (A) Position of the T-DNA insertion in *anac060* (Col accession). Black bars indicate the protein coding exons, open bars indicate the introns and the gray bars indicate the 5′ and 3′ UTRs. LP, RP and LB indicate the PCR primers used to confirm the insertion. (B) PCR confirmation of the T-DNA insertion. The PCR product of LB+RP indicates the presence of the T-DNA insertion. The PCR product of LP+RP indicates the absence of the insertion in Col. Primer sequences were: LB, 5′-ATTTTGCCGATTTCGGAAC-3′; LP, 5′-TGGACTCTGTTTGAAGCCTTG-3′; RP, 5′-TATGCCTGTCCTGATTTGCTC -3′. (C) Expression analysis of *ANAC060* in Col and Salk_012554C using RT-PCR. Primers: ANAC060-F, 5′-AGGAGGAAGAACGGAATGGCTT-3′; ANAC060-R, 5′-GGACTCTGTTTGAAGCCTTGGTAC-3′; PP2A-F, 5′-AAGGTAAAGAAGACAGCAACGA-3′; PP2A-R, 5′-CAAAAAGCAAATACGCCC-3′.
(TIF)

Figure S2 Fructose sensitivity phenotypes of Col (A), *anac060* (B), and *ANAC060* (Col) transgenic complementation of the *anac060* mutant (C). Seeds were plated on 1/2 MS containing 6.5% fructose and grown for 9 days at 22°C under continuous illumination.
(TIF)

Figure S3 Transcriptional activity in yeast of different Col *GSQ11/ANAC060* constructs as indicated. GAL4 AD, GAL4 activation domain; NLS, nuclear localization sequence; MCS, multiple cloning site. Dilutions as indicated of transformed PJ69-4A yeast cells were grown on selective medium (SD/-Trp -His -Ade) and compared to growth on non-selective control medium (SD/-Trp).
(TIF)

Figure S4 The QTN affects the splicing pattern of *ANAC060* mRNA. (A) cDNA sequence of *ANAC060* mRNA expressed in transgenic *anac060* transformed with mutated Col *ANAC060* ^G-T^. The red line indicated the exon 3 acceptor splice site. (B) cDNA sequence of *ANAC060* mRNA expressed in transgenic *anac060* transformed with Col *ANAC060*. The red line indicates the exon 3 acceptor splice site, the extra 20 bp are located between the red and the black lines.
(TIF)

Figure S5 Glc sensitivity phenotypes of Col (A), and *35S-ABI4::GFP* lines (B). Seeds were plated on 1/2 MS containing 5.5% Glc and grown for 9 days at 22°C under continuous illumination. (C) *ANAC060* mRNA expression levels of 7-day-old Col and *35S-ABI4::GFP* seedlings grown on 5.5% Glc. The values represent the average of three technical repeats from a representative experiment. The bars indicate the standard errors. Similar results were obtained in three independent experiments.
(TIF)

Figure S6 Col, *anac060* and NIL N40 seedlings grown on 1/2 MS control medium for 5 d under continuous illumination.
(TIF)

Table S1 Monogenic segregation of SSLP markers on Chromosome 3.
(DOCX)

Table S2 The QTN haplotypes of the accessions as determined by CAPS marker.
(DOCX)

Table S3 The QTN haplotypes of the Arabidopsis 1001 database (http://signal.salk.edu/atg1001/3.0/gebrowser.php).
(DOCX)

Table S4 Primers used for cloning promoter and coding region of the Col *ANAC060* gene.
(DOCX)

Table S5 Primers used for cloning the coding region of Col and C24 *ANAC060* genes.
(DOCX)

Table S6 Primers used for the transactivation activity assay.
(DOCX)

Table S7 Primers used for *ANAC060* promoter ChIP-qPCR.
(DOCX)

Table S8 Primers used for cloning the P3 fragment of Col *ANAC060* promoter.
(DOCX)

Table S9 Primers used for qPCR.
(DOCX)
