# Supplementary figures and images for: The ABI4-Induced Arabidopsis ANAC060 Transcription Factor Attenuates ABA Signaling and Renders Seedlings Sugar Insensitive when Present in the Nucleus
Source: PLoS Genet. 2014 Mar 13;10(3):e1004213. doi: 10.1371/journal.pgen.1004213 (PMC3953025; doi:10.1371/journal.pgen.1004213)

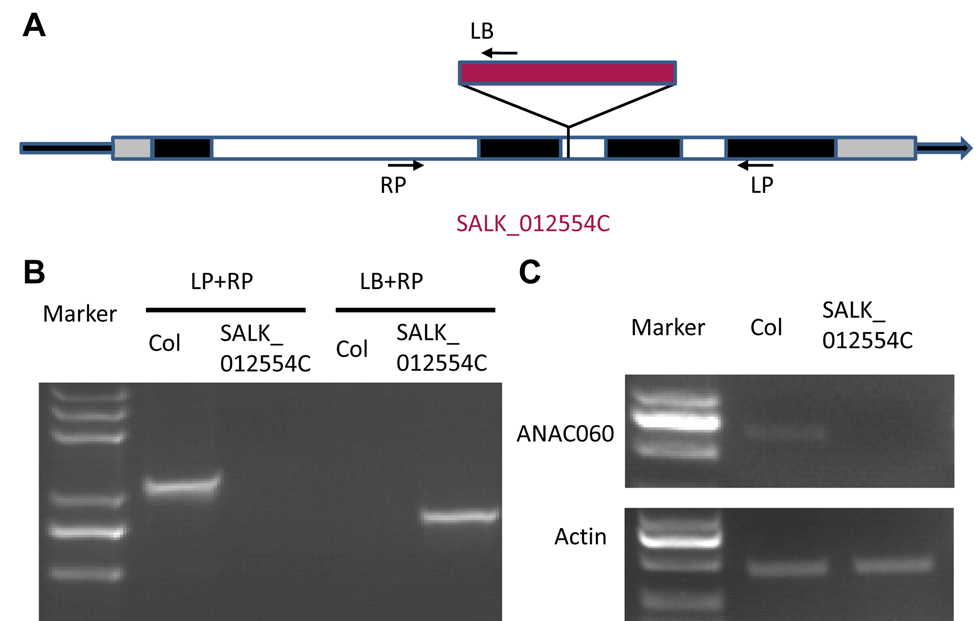

Supplement: Figure S1 — Confirmation of the anac060 T-DNA insertion mutant (Salk_012554C). (A) Position of the T-DNA insertion in anac060 (Col accession). Black bars indicate the protein coding exons, open bars indicate the introns and the gray bars indicate the 5′ and 3′ UTRs. LP, RP and LB indicate the PCR primers used to confirm the insertion. (B) PCR confirmation of the T-DNA insertion. The PCR product of LB+RP indicates the presence of the T-DNA insertion. The PCR product of LP+RP indicates the absence of the insertion in Col. Primer sequences were: LB, 5′-ATTTTGCCGATTTCGGAAC-3′; LP, 5′-TGGACTCTGTTTGAAGCCTTG-3′; RP, 5′-TATGCCTGTCCTGATTTGCTC -3′. (C) Expression analysis of ANAC060 in Col and Salk_012554C using RT-PCR. Primers: ANAC060-F, 5′-AGGAGGAAGAACGGAATGGCTT-3′; ANAC060-R, 5′-GGACTCTGTTTGAAGCCTTGGTAC-3′; PP2A-F, 5′-AAGGTAAAGAAGACAGCAACGA-3′; PP2A-R, 5′-CAAAAAGCAAATACGCCC-3′. (TIF) [file pgen.1004213.s001.tif]

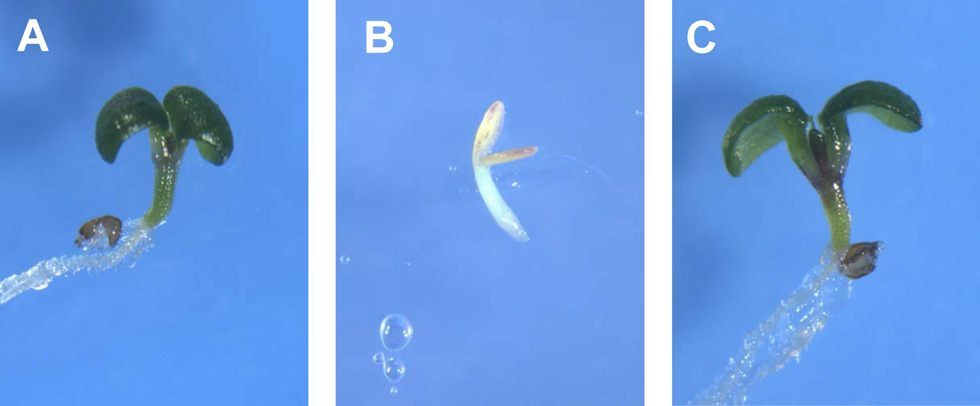

Supplement: Figure S2 — Fructose sensitivity phenotypes of Col (A), anac060 (B), and ANAC060 (Col) transgenic complementation of the anac060 mutant (C). Seeds were plated on 1/2 MS containing 6.5% fructose and grown for 9 days at 22°C under continuous illumination. (TIF) [file pgen.1004213.s002.tif]

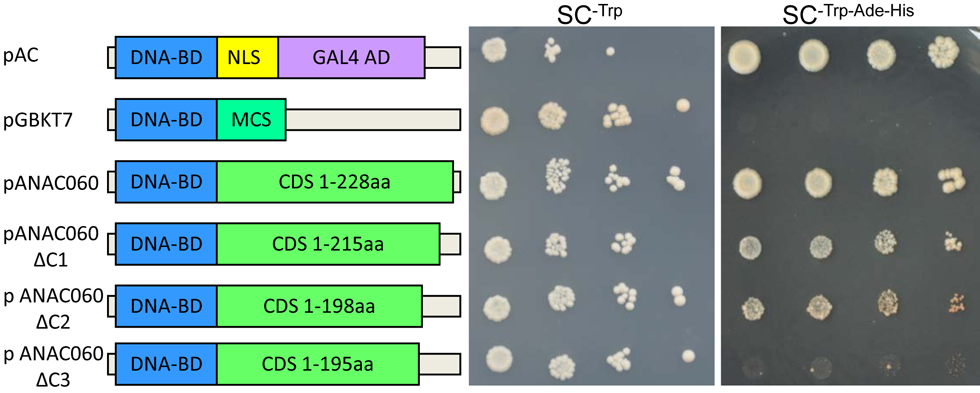

Supplement: Figure S3 — Transcriptional activity in yeast of different Col GSQ11/ANAC060 constructs as indicated. GAL4 AD, GAL4 activation domain; NLS, nuclear localization sequence; MCS, multiple cloning site. Dilutions as indicated of transformed PJ69-4A yeast cells were grown on selective medium (SD/-Trp -His -Ade) and compared to growth on non-selective control medium (SD/-Trp). (TIF) [file pgen.1004213.s003.tif]

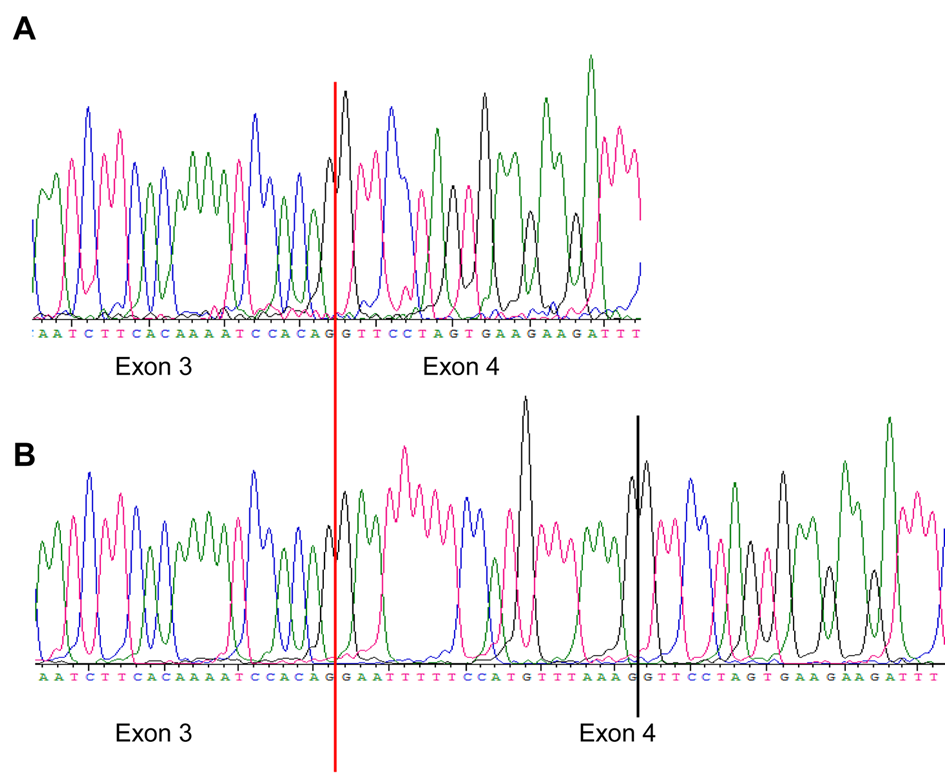

Supplement: Figure S4 — The QTN affects the splicing pattern of ANAC060 mRNA. (A) cDNA sequence of ANAC060 mRNA expressed in transgenic anac060 transformed with mutated Col ANAC060 G-T. The red line indicated the exon 3 acceptor splice site. (B) cDNA sequence of ANAC060 mRNA expressed in transgenic anac060 transformed with Col ANAC060. The red line indicates the exon 3 acceptor splice site, the extra 20 bp are located between the red and the black lines. (TIF) [file pgen.1004213.s004.tif]

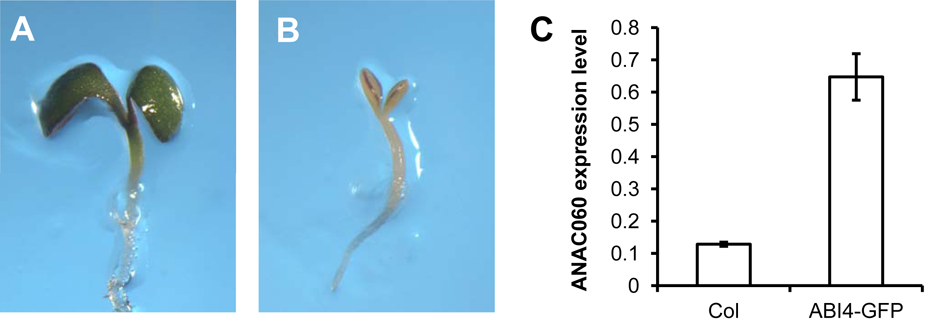

Supplement: Figure S5 — Glc sensitivity phenotypes of Col (A), and 35S-ABI4::GFP lines (B). Seeds were plated on 1/2 MS containing 5.5% Glc and grown for 9 days at 22°C under continuous illumination. (C) ANAC060 mRNA expression levels of 7-day-old Col and 35S-ABI4::GFP seedlings grown on 5.5% Glc. The values represent the average of three technical repeats from a representative experiment. The bars indicate the standard errors. Similar results were obtained in three independent experiments. (TIF) [file pgen.1004213.s005.tif]

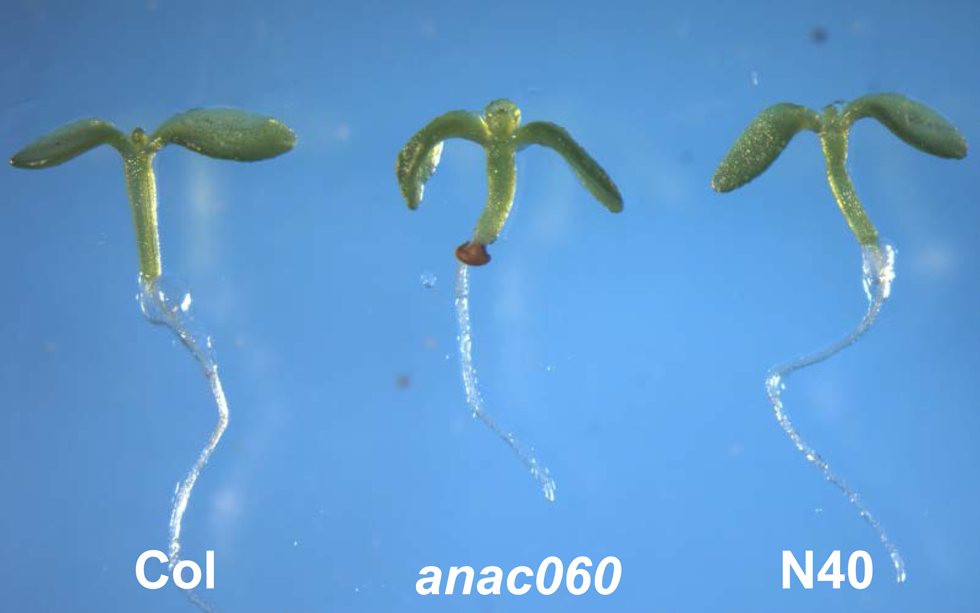

Supplement: Figure S6 — Col, anac060 and NIL N40 seedlings grown on 1/2 MS control medium for 5 d under continuous illumination. (TIF) [file pgen.1004213.s006.tif]
