# Supplementary material for: The ABI4-Induced Arabidopsis ANAC060 Transcription Factor Attenuates ABA Signaling and Renders Seedlings Sugar Insensitive when Present in the Nucleus
Source: PLoS Genet. 2014 Mar 13;10(3):e1004213. doi: 10.1371/journal.pgen.1004213 (PMC3953025; doi:10.1371/journal.pgen.1004213)
Supplement: Table S1 — Monogenic segregation of SSLP markers on Chromosome 3. (DOCX) [file pgen.1004213.s007.docx]

Table S1. Monogenic segregation of SSLP markers on Chromosome 3

|  | Selected | Population |  |  |  | Unselected | Population |  |  |
| --- | --- | --- | --- | --- | --- | --- | --- | --- | --- |
|  | Genotype |  |  |  |  | Genotype |  |  |  |
|  | Col/Col | Col/C24 | C24/C24 | χ^2^(1:2:1) |  | Col/Col | Col/C24 | C24/C24 | χ^2^ (1:2:1) |
| NGA32 | 25 | 48 | 26 | 0.106 |  | 25 | 41 | 18 | 1.220 |
| NGA172 | 22 | 50 | 28 | 0.745 |  | 26 | 42 | 22 | 0.717 |
| ATHCHIB2 | 27 | 45 | 29 | 1.193 |  | 25 | 44 | 20 | 0.590 |
| NT204 | 28 | 44 | 30 | 1.887 |  | 23 | 47 | 19 | 0.725 |
| MSAT3.19 | 21 | 49 | 18 | 1.483 |  | 24 | 43 | 16 | 1.719 |
| MSAT3.23 | 20 | 45 | 14 | 2.614 |  | 27 | 41 | 14 | 4.152* |
| F7K15 | 31 | 55 | 16 | 5.142* |  | 26 | 49 | 18 | 1.726 |
| F13I12 | 30 | 55 | 16 | 4.797* |  | 25 | 49 | 20 | 0.771 |
| CDC2BG | 34 | 49 | 19 | 4.554* |  | 25 | 43 | 18 | 1.169 |
| NGA112 | 28 | 47 | 14 | 4.770* |  | 21 | 44 | 18 | 0.608 |
