# Supplementary material for: The ABI4-Induced Arabidopsis ANAC060 Transcription Factor Attenuates ABA Signaling and Renders Seedlings Sugar Insensitive when Present in the Nucleus
Source: PLoS Genet. 2014 Mar 13;10(3):e1004213. doi: 10.1371/journal.pgen.1004213 (PMC3953025; doi:10.1371/journal.pgen.1004213)
Supplement: Table S2 — The QTN haplotypes of the accessions as determined by CAPS marker. (DOCX) [file pgen.1004213.s008.docx]

Table S2. The QTN haplotypes of the accessions as determined by CAPS marker.

| Haplotypes | Accessions |
| --- | --- |
| G (Col type) | Hn-0 , Kelsterbach-2 , Kro-0 , Li-3 , Or-0 , Paw-3 |
| T (C24 type) | Ag-0 , ALL1-3 , App1-16 , Br-0 , Can-0 , Fja1-2 , Gal1-2 , Goettingen-7 , Hau-0 , Hey-1 , Je-0 , JI-3 , KI-5 , Kn-0 , Knox-11 , Kr-0 , Krot-2 , Li-6 , Lis-2 , Mc-0 , MNF-Che-2 , MNF-Jac-32 , MNF-Pot-68 , Mnz-0 , Mr-0 , N4 , N7 , NC-6 , NFC20 , No-0 , Nok-1 , Nw-0 , Nw-2 , Ob-1 , Omo2-1 , Pa-2 , PAR-5 , Pent-1 , PHW-14 , PHW-20 , PHW-26 , PHW-28 , PHW-33 , PHW-35 , PHW-36 , Pla-0 , Pog-0 , Rev-2 , ROM-1 , Sparta-1 , T1040 , T1060 , T1080 , T1110 , UKNW06-436 , UKNW06-460 , UKSE-062 , UKSE-192 , UKSW06-202 |
