# Supplementary material for: The ABI4-Induced Arabidopsis ANAC060 Transcription Factor Attenuates ABA Signaling and Renders Seedlings Sugar Insensitive when Present in the Nucleus
Source: PLoS Genet. 2014 Mar 13;10(3):e1004213. doi: 10.1371/journal.pgen.1004213 (PMC3953025; doi:10.1371/journal.pgen.1004213)
Supplement: Table S3 — The QTN haplotypes of the Arabidopsis 1001 database (http://signal.salk.edu/atg1001/3.0/gebrowser.php). (DOCX) [file pgen.1004213.s009.docx]

Table S3. The QTN haplotypes of the Arabidopsis 1001 database (http://signal.salk.edu/atg1001/3.0/gebrowser.php)

| Haplotypes | Accessions |
| --- | --- |
| G (Col type) | Ak-1, Alst-1, Altai-5, Amel-1, Appt-1, Baa1-2, Baa4-1, Baa5-1, Bay-0, Bch-1, Bsch-0, Ca-0, Chi-0, Col-0, Dr-0, Ei-2, En-2, Est, Fr-2, Gel-1, Ha-0, Hn-0, Hov1-10, Hovdala-2, Is-0, Kelsterbach-4, Kent, Kil-0, Koln, Kondara, Kro-0, Kro-0, Kyoto, Lan-0, Le-0, Lisse, Lm-2, Mz-0, NFA-10, Nd-1, Nz-1, Or-0, Oy-0, Oy-0, Per-1, Pna-17, Po-0, Pro-0, Ragl-1, Ri-0, Roed-17-319, Rue3-1-31, Sakata, San-2, Sq-8, Stw-0, TAAL-07, Tottarp-2, Tsu-0, Tsu-1, Tu-0, TueSB30-3, Utrecht, Vaar2-1, Wa-1, WalhaesB4, Wil-1, Wl-0, Ws-2 |
| T (C24 type) | Aa-0, Abd-0, Aedal-1, Aedal-3, Ag-0, Agu-1, Alc-0, Ale-Stenar-44-4, Ale-Stenar-56-14, Ale-Stenar-64-24, Algutsrum, An-1, Ang-0, Anholt-1, Ann-1, Anz-0, App1-12, App1-14, App1-16, Ba-1, Baa-1, Bak-2, Bak-7, Bd-0, Benk-1, Ber, Bg-2, Bik-1, Bil-5, Bil-7, Bl-1, Bla-1, Blh-1, Boot-1, Bor-1, Bor-4, Br-0, Broesarp-34-145, Broet1-6, Bs-1, Bu-0, Buckhorn-Pass, Bur-0, Bur-0, C24, Cal-0, Can-0, Cdm-0, Cerv-1, Chat-1, CIBC-17, CIBC-5, Cnt-1, Co, Co-1, Com-1, Ct-1, Cvi-0, Cvi-0, Da1-12, Db-1, Del-10, Dem-4, Di-G, Dja-1, Do-0, Doer-10, Dog-4, Don-0, Dra-0, Dra2-1, Dra3-1, DraII-1, DraIII-1, Duk, Durh-1, Eden-1, Eden-2, Eden-7, Eden-9, Edi-0, Eds-1, Eds-9, El-0, Ema-1, En-1, En-D, Er-0, Es-0, Est-1, Et-0, Etna-2, Ey15-2, Faeb-2, Faeb-4, Fael-1, Fei-0, Fi-0, Fjae1-1, Fjae1-2, Fjae1-5, Fjae2-4, Fly2-1, Fly2-2, Fri-2, Ga-0, Ge-0, Gie-0, Gifu-2, Gr-1, Gre-0, Gro-3, Groen-12, Groen-14, Groen-5, Gu-0, Gy-0, Had-1, Had-2, Hag-2, Hal-1, Ham-1, Hau-0, Hel-3, Hey-1, Hh-0, Hi-0, HKT2.4, Hod, Hola-1-2, Hola-1-1, Hola-2-2, Hov1-7, Hov3-2, Hov3-5, Hov4-1, HR-10, HR-5, Hs-0, HSm, ICE1, ICE102, ICE104, ICE106, ICE107, ICE111, ICE112, ICE119, ICE120, ICE127, ICE130, ICE134, ICE138, ICE150, ICE152, ICE153, ICE163, ICE169, ICE173, ICE181, ICE21, ICE212, ICE213, ICE216, ICE226, ICE228, ICE29, ICE33, ICE36, ICE49, ICE50, ICE60, ICE61, ICE63, ICE7, ICE70, ICE71, ICE72, ICE73, ICE75, ICE79, ICE91, ICE92, ICE93, ICE97, ICE98, In-0, Istisu-1, Je-0, Jea, Jl-3, Jm-0, Kaevlinge-1, Kal-2, Kar-1, Kas-1, Kas-2, Kastel-1, Kb-0, Kia-1, Kin-0, Kl-5, Kn-0, Kni-1, Knox-18, Ko-2, Koch-1, Kor-3, Krot-0, Kru-3, Kulturen-1, Kz-9, La-0, Lag2.2, Lan-1, Leo-1, Ler-0, Ler-1, Ler-1, Lerik1-3, Li-2:1, Liarum, Lilloe-1, Lip-0, Lis-2, Lis-3, Litva, LL-0, Lom1-1, love-1, love-5, Lp2-2, Lp2-6, Lu-1, Lund, Mc-0, Mer-6, Mh-0, Mir-0, Mnz-0, Ms-0, Mt-0, Mv-0, N13, Naes-2, Nc-1, NC-6, Nemrut-1, Neo-6, NFA-8, Nie1-2, No-0, Nok-3, Np-0, Nw-0, Nyl-13, Nyl-2, Nyl-7, Ob-0, Oede-2, Oemoe1-7, Oemoe2-1, Oer-1, Old-1, Omn-1, Omn-5, Ove-0, Ped-0, PHW-2, PHW-34, Pi-0, Pla-0, Pna-10, Pog-0, Pra-6, Pt-0, Pu2-23, Pu2-7, Pu2-8, Puk-2, Qar-8a, Qui-0, Ra-0, Rak-2, Rd-0, Ren-1, Ren-11, Rennes-1, Rev-1, Rev-2, Rhen-1, Rld-1, Rmx-A02, Rmx-A180, Rome-1, Rou-0, RRs-10, RRS-7, Rsch-4, Rubeznhoe-1, Sanna-2, Sap-0, Se-0, Seattle-0, Sei-0, Sf-1, Sf-2, Sg-1, Sha, Sha, Si-0, Sim-1, Sorbo, Sp-0, Sparta-1, Spr1-2, Spr1-6, Spro-1, Spro-2, Spro-3, Sq-1, Sr:3, Sr:5, St-0, Star-8, Ste-2, Ste-3, Ste-4, Stu1-1, Su-0, Sus-1, T1000, T1020, T1070, T1080, T1090, T1110, T1130, T1160, T460, T470, T480, T530, T540, T550, T570, T710, T720, T740, T780, T790, T800, T840, T850, T860, T880, T900, T930, T960, T980, T990, Ta-0, TAA-04, TAA-14, TAA-18, TAAD-01, TAAD-03, TAAD-04, TAAD-05, TAAD-06, TAAL-03, Tamm-2, Tamm-27, Tamm-2, TBO-01, TDr-1, TDr-13, TDr-16, TDr-17, TDr-2, TDr-7, TDr-8, TDr-9, TEDEN-02, TEDEN-03, TFAE-06, TFAE-07, TFAE-08, Tgr-01, Tha-1, Ting-1, Tny-04, Tol-0, TOM-04, TOM-06, TOM-07, Tomegap-2, TRAE-01, Ts-1, Ts-5, Tscha-1, Tuescha9, TueV13, TueWa1-2, Tul-0, Tur-4, TV-10, TV-22, TV-30, TV-38, TV-7, Ty-0, Uk-1, Ull2-3, Ull2-5, Ull-A-1, Uod-1, Uod-7, Vaar-1, Vaar2-6, Vaestervik, Van-0, Vash-1, Ven-1, Vie-0, Vimmerby, Vind-1, Vinsloev, Wc-1, Wei-0, Westkar-4, Wil-2, Ws-0, Wt-5, Wu-0, Xan-1, Yeg-1, Yo-0, Yst-1, Zal-1, Zdr-1, Zu-0 |
| C | Got-22, Got-7 |
