# Supplementary material for: The ABI4-Induced Arabidopsis ANAC060 Transcription Factor Attenuates ABA Signaling and Renders Seedlings Sugar Insensitive when Present in the Nucleus
Source: PLoS Genet. 2014 Mar 13;10(3):e1004213. doi: 10.1371/journal.pgen.1004213 (PMC3953025; doi:10.1371/journal.pgen.1004213)
Supplement: Table S4 — Primers used for cloning promoter and coding region of the Col ANAC060 gene. (DOCX) [file pgen.1004213.s010.docx]

Table S4. Primers used for cloning promoter and coding region of the Col *ANAC060* gene

|  | Forward | Reverse |
| --- | --- | --- |
| Promoter | ggaattccTCGTACATCCGCCTAAATAGACG | ggggtaccccAAGGGTAGAGATGATGCAAAGAG |
| Codon | cgggatcccgATGGCAGCTGCACCACCGATC | aactgcagaaccaatgcattgg TCACACTAAATAAAACACCAAC |

* The lower case letters are the extra sequences for restriction enzyme site insertions.
