# Supplementary material for: The ABI4-Induced Arabidopsis ANAC060 Transcription Factor Attenuates ABA Signaling and Renders Seedlings Sugar Insensitive when Present in the Nucleus
Source: PLoS Genet. 2014 Mar 13;10(3):e1004213. doi: 10.1371/journal.pgen.1004213 (PMC3953025; doi:10.1371/journal.pgen.1004213)
Supplement: Table S6 — Primers used for the transactivation activity assay. (DOCX) [file pgen.1004213.s012.docx]

Table S6. Primers used for the transactivation activity assay

|  | Forward | Reverse |
| --- | --- | --- |
| pANAC060 | cgggatcccgATGGCAGCTGCACCACCG | aactgcagaaccaatgcattgg TTAAACATGGAAAAATTCCTGT |
| pANAC060ΔC1 | cgggatcccgATGGCAGCTGCACCACCG | aactgcagaaccaatgcattggAGATTCTGCTATTTCACTAAG |
| pANAC060ΔC2 | cgggatcccgATGGCAGCTGCACCACCG | aactgcagaaccaatgcattggGAAATCAACCATAGTTTCCCA |
| pANAC060ΔC3 | cgggatcccgATGGCAGCTGCACCACCG | aactgcagaaccaatgcattggCATAGTTTCCCAACCATAAATAC |

* The lower case letters are the extra sequences for restriction enzyme site insertions.
