# Supplementary material for: The ABI4-Induced Arabidopsis ANAC060 Transcription Factor Attenuates ABA Signaling and Renders Seedlings Sugar Insensitive when Present in the Nucleus
Source: PLoS Genet. 2014 Mar 13;10(3):e1004213. doi: 10.1371/journal.pgen.1004213 (PMC3953025; doi:10.1371/journal.pgen.1004213)
Supplement: Table S7 — Primers used for ANAC060 promoter ChIP-qPCR. (DOCX) [file pgen.1004213.s013.docx]

Table S7. Primers used for *ANAC060* promoter ChIP-qPCR

|  | Forward | Reverse | | |
| --- | --- | --- | --- | --- |
| P1 | TGTTGATCCGTCCAAAGTTAGT | | GGAAATAAAACGTCTTTTCAGGA |  |
| P2 | GAGGCAGGAGTAGTAACGTGG | | TCACTCACACCTGCGTTTCAGT | |
