# Supplementary material for: The ABI4-Induced Arabidopsis ANAC060 Transcription Factor Attenuates ABA Signaling and Renders Seedlings Sugar Insensitive when Present in the Nucleus
Source: PLoS Genet. 2014 Mar 13;10(3):e1004213. doi: 10.1371/journal.pgen.1004213 (PMC3953025; doi:10.1371/journal.pgen.1004213)
Supplement: Table S8 — Primers used for cloning the P3 fragment of Col ANAC060 promoter. (DOCX) [file pgen.1004213.s014.docx]

Table S8. Primers used for cloning the P3 fragment of Col *ANAC060* promoter

|  | Forward | Reverse |
| --- | --- | --- |
| P3 | ccgctcgagcggGATTTTGTTGATCCGTCCA | aactgcagaaCGTGTTTATTAATTTATCGAG |

* The lower case letters are the extra sequences for restriction enzyme site insertions.
