# Supplementary material for: The ABI4-Induced Arabidopsis ANAC060 Transcription Factor Attenuates ABA Signaling and Renders Seedlings Sugar Insensitive when Present in the Nucleus
Source: PLoS Genet. 2014 Mar 13;10(3):e1004213. doi: 10.1371/journal.pgen.1004213 (PMC3953025; doi:10.1371/journal.pgen.1004213)
Supplement: Table S9 — Primers used for qPCR. (DOCX) [file pgen.1004213.s015.docx]

Table S9. Primers used for qPCR

| Gene | Forward | | | Reverse |
| --- | --- | --- | --- | --- |
| PP2A | TAACGTGGCCAAAATGATGC | | GTTCTCCACAACCGCTTGGT | |
| ANAC060 | AACTTCGAGCCTTGGGATTT | CGTGGGGATACTTCTTACCG | | |
| ABI4 | CCGCTTCTTCTCCTTCCAC | GAGGGAGGAGAGGTCTTAGGG | | |
